# Supplementary material for: Application of slow-controlled release fertilizer coordinates the carbon flow in carbon-nitrogen metabolism to effect rice quality
Source: BMC Plant Biol. 2024 Jun 29;24:621. doi: 10.1186/s12870-024-05309-9 (PMC11218275; doi:10.1186/s12870-024-05309-9)
Supplement: Supplementary file 1 — Supplementary Material 1. [file 12870_2024_5309_MOESM1_ESM.pdf]

Table S1. Sequences of primers for Actin and genes for qRT-PCR.

Gene	Forward primer 5′ → 3’	Reverse primer 5′ → 3’
Actin	CAATCGTGAGAAGATGACCC	GTCCATCAGGAAGCTCGTAGC
OsSUT2	TCTTTTATCGGTGGGCTGGT	TTGCAAAGAATGGCCGACAA
OsCIN4	CATGTTTGTGCCGGATACTG	TGCCATCACCATCTAACCAA
OsGS2	AGGATCGGACAAATCGTTTGG	GCATGACCTCTCCATTTGTTCC

Table S2. Principal components analysis about the effect of slow-controlled release fertilizer on key enzymes activities related carbon-nitrogen metabolism in rice grain at filling stage in 2020 and 2021.

Traits	PC1	PC2	PC3	PC4	PC5	PC6
SuSase	0.44	-0.24	-0.12	-0.34	0.61	-0.50
ADPGase	0.41	-0.42	0.17	0.61	-0.37	-0.34
GBSS	0.44	-0.22	0.31	0.07	0.31	0.75
SBE	0.44	-0.09	-0.32	-0.56	-0.61	0.14
GS	0.37	0.53	-0.61	0.43	0.14	0.10
GOGAT	0.33	0.66	0.62	-0.13	-0.11	-0.20
Eigenvalue	4.62	0.88	0.28	0.11	0.08	0.03
Contributive ratio (%)	76.93	14.74	4.66	1.86	1.27	0.54
Cumulative (%)	76.93	91.67	96.33	98.19	99.46	100.00

Table S3. Principal components analysis of comprehensive index and loading matrix of each component for amino acids accumulation under the application of slow-controlled release fertilizers in 2020 and 2021.

Traits	PC1	PC2	PC3	PC4	PC5	PC6	PC7	PC8	PC9	PC10	PC11	PC12
Asp	0.30	0.13	0.14	-0.49	0.22	-0.16	0.35	0.28	-0.16	-0.57	-0.08	-0.05
Thr	0.28	-0.39	0.08	0.08	0.00	0.62	-0.09	0.17	-0.50	-0.04	0.30	0.06
Ser	0.25	0.31	-0.47	0.19	0.21	0.48	0.17	0.08	0.50	-0.13	0.05	0.06
Glu	0.34	0.26	-0.02	-0.24	0.17	0.04	0.28	-0.07	-0.25	0.73	-0.21	0.01
Gly	0.35	-0.08	0.09	-0.21	-0.09	0.17	-0.32	-0.49	0.16	-0.11	-0.22	-0.59
Val	0.36	-0.14	0.12	-0.20	-0.10	-0.04	-0.27	-0.20	0.23	-0.04	-0.17	0.77
Ile	0.32	-0.32	0.09	0.01	0.06	-0.26	-0.19	0.61	0.40	0.28	0.13	-0.22
Leu	0.30	0.39	-0.15	-0.04	-0.28	-0.29	-0.15	-0.15	-0.12	-0.01	0.71	-0.01
Tyr	0.27	-0.02	-0.55	0.31	-0.24	-0.26	-0.16	0.17	-0.36	-0.13	-0.46	-0.02
Phe	0.13	0.45	0.56	0.31	-0.48	0.19	0.04	0.24	0.04	-0.04	-0.21	-0.02
Lys	0.20	0.14	0.29	0.52	0.67	-0.21	-0.20	-0.17	-0.13	-0.09	0.02	0.02
His	0.26	-0.40	0.08	0.33	-0.21	-0.18	0.68	-0.31	0.13	-0.03	0.08	-0.02
Eigenvalue	6.57	1.47	0.99	0.89	0.74	0.47	0.29	0.19	0.19	0.08	0.07	0.04
Contributive ratio (%)	54.75	12.25	8.29	7.44	6.19	3.93	2.45	1.59	1.55	0.70	0.55	0.32
Cumulative (%)	54.75	67.00	75.29	82.73	88.92	92.84	95.29	96.89	98.44	99.13	99.68	100.00

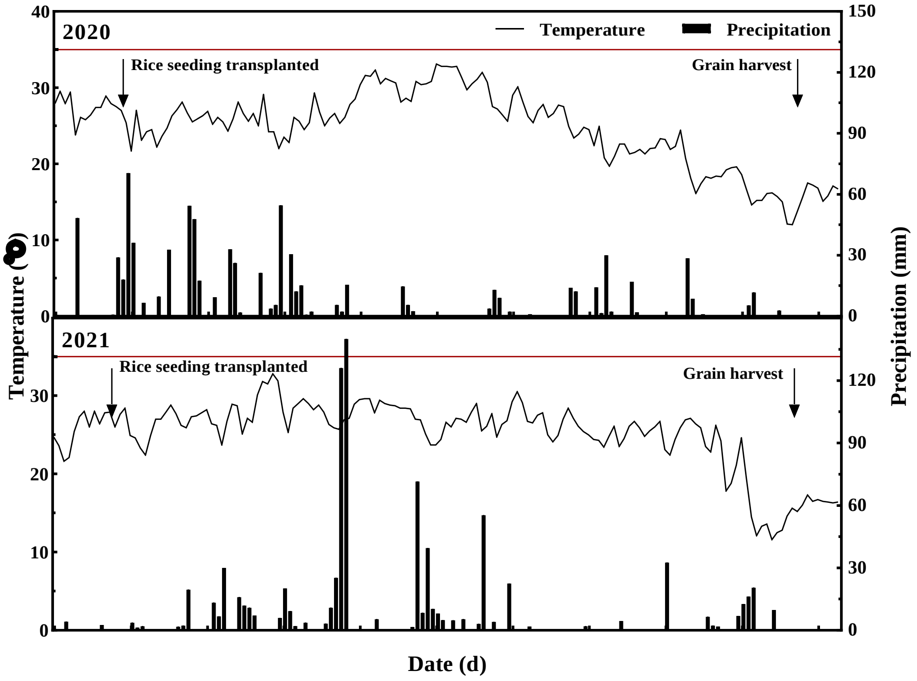

Figure S1. Daily temperature (line) and precipitation (bars) during the rice growth seasons in 2020 and 2021.

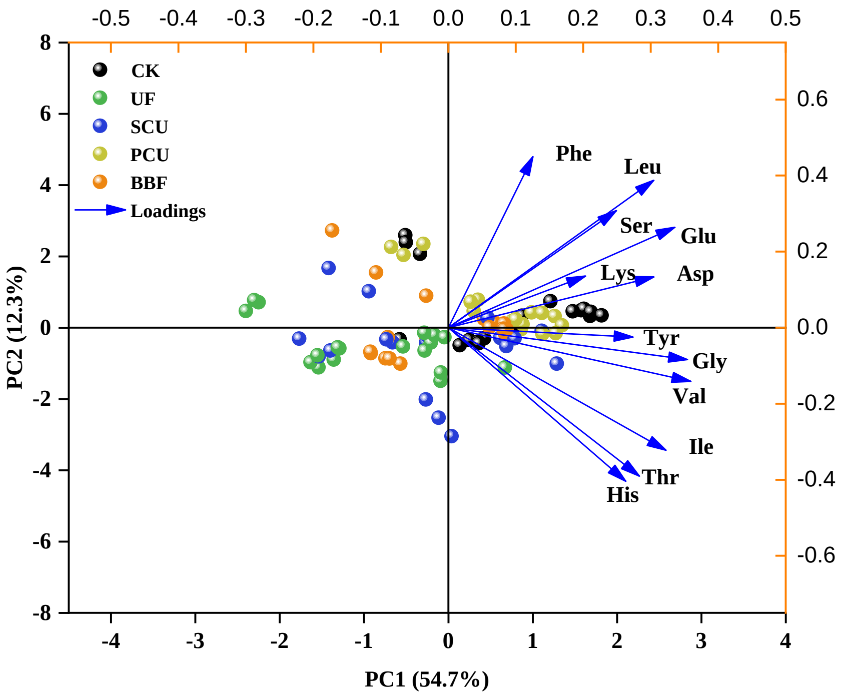

Figure S2. Principal component analysis about the effect of slow-controlled release fertilizer application on the accumulation of amino acids in rice grains at the filling stage in 2020 and 2021. CK, conventional fertilization with four spilt applications of urea; UF, urea formaldehyde; SCU, sulfur-coated urea; PCU, polymer-coated urea; BBF, controlled-release bulk blending fertilizer.
